# Physical Activity in the Prevention and Treatment of Stroke

**DOI:** 10.5402/2011/953818

**Published:** 2011-10-01

**Authors:** Siobhan Gallanagh, Terry J. Quinn, Jen Alexander, Matthew R. Walters

**Affiliations:** ^1^School of Medicine, University of Glasgow, Glasgow G12 8QQ, UK; ^2^Institute of Cardiovascular and Medical Sciences, University of Glasgow, Glasgow G11 6NT, UK; ^3^Department of Physiotherapy, Western Infirmary, Glasgow G11 6NT, UK

## Abstract

The role of physical activity in the prevention of stroke is of great interest due to the high mortality and significant impact of stroke-related morbidity on the individual and on healthcare resources. The use of physical activity as a therapeutic strategy to maximise functional recovery in the rehabilitation of stroke survivors has a growing evidence base. This narrative review examines the existing literature surrounding the use of exercise and physical therapy in the primary and secondary prevention of stroke. It explores the effect of gender, exercise intensities and the duration of observed benefit. It details the most recent evidence for physical activity in improving functional outcome in stroke patients. The review summaries the current guidelines and recommendations for exercise therapy and highlights areas in which further research and investigation would be useful to determine optimal exercise prescription for effective prevention and rehabilitation in stroke.

## 1. Introduction

Stroke is a leading cause of mortality and morbidity worldwide. In the UK stroke is the third most common cause of death and the main cause of acquired disability. Approximately 130,000 individuals experience a first ever stroke per annum [1]. In addition to widely applicable pharmacological treatment for acute stroke, effective prevention and rehabilitation strategies are crucial. The development of such strategies is a major challenge for the 21st century medicine. 

Exercise and physical activity have an increasing evidence base in the primary and secondary prevention of stroke and in stroke rehabilitation. The interface between physical activity and cerebrovascular disease is complex and of broad interest to clinicians, therapists, and epidemiologists. The importance of the relationship is becoming clearer: physical inactivity has been implicated by the INTERSTROKE study as one of the 5 key risk factors which account for more than 80% of the global burden of stroke [2]. Physical fitness training is increasingly being recommended as a component of stroke rehabilitation programmes due to the emerging body of evidence surrounding the benefits in improving the function after stroke [3]. The role of long-term physical activity in patients who have had a stroke in the prevention of further stroke is less clear. This paper provides a narrative review of the literature which addresses the interface between physical activity and cerebrovascular disease with specific reference to prevention of stroke and poststroke rehabilitation.

## 2. Search Strategy

A computer-assisted literature search was performed using the database Ovid MEDLINE (1950–June Week 4 2010) and the database EMBASE (1980 to 2010 Week 25). Briefly, we sought to identify studies examining the relationship between exercise or physical activity and cerebrovascular risk and studies examining the effect of exercise or physical activity in populations with cerebrovascular disease. Reference lists of all identified relevant articles and reviews were screened to identify other potentially eligible studies.

## 3. Effect of Exercise on Modifiable Cardiovascular Risk Factors

Hypertension is recognised as the most important modifiable risk factor for both ischaemic and haemorrhagic stroke [4]. A strong and well-recognised relationship exists between blood pressure and stroke risk [5]. Physical activity is associated with reductions in blood pressure and in the risk of developing hypertension in healthy normotensive individuals [6–8], thus positively altering a major contributor to stroke risk. 

**Figure 1:**
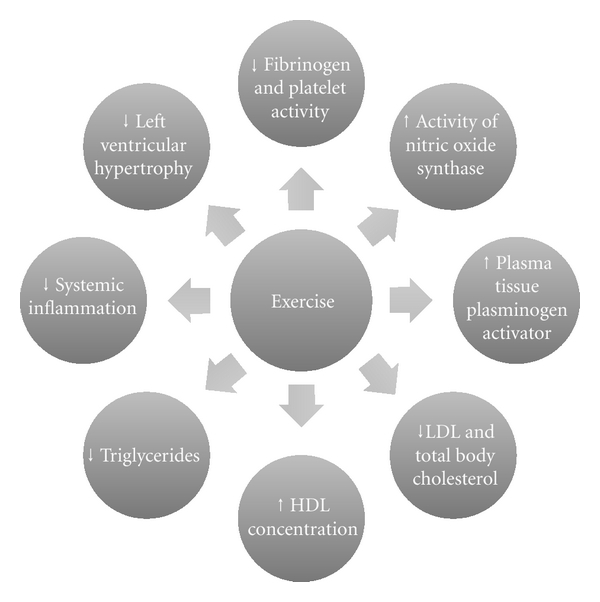
Putative pathophysiological benefits of exercise [9–15].

The mechanistic basis of the effect of exercise on stroke risk is likely to be multifactorial. Regular exercise is known to increase the activity of nitric oxide synthase improving endothelial function, reduce left ventricular hypertrophy; stimulate elevations in plasma tissue plasminogen activator and HDL concentrations, and reduce fibrinogen and platelet activity. Aerobic conditioning has been shown to enhance glucose regulation and promote reductions in total serum and LDL cholesterol, triglycerides, total body fat, and systemic inflammation (Figure 1) [9–15]. Therefore, among other mechanisms, exercise helps to prevent obesity, hypertension, dyslipidaemia, and the development of type 2 diabetes, all of which are implicated in the pathogenesis of stroke.

## 4. Physical Activity in Primary Prevention of Stroke

Exercise and physical activity have a well-established evidence base for their benefits in reducing cardiovascular risk factors through the mechanisms described above. Observational studies have found an inverse association between physical activity and stroke risk [16–30], with recent reviews estimating that physical activity is associated with a 25–30% risk reduction for stroke [31], however, this finding has not been consistently reproduced in the literature, with other groups reporting U-shaped associations or no associations at all [32–37] (Table 1). 

Differences in methodology, patient populations, and exercise interventions are likely to have contributed to this disparity. Studies generally, have been limited by their observational design and variations in adjustment for residual confounding. Some of the studies are retrospective and case control, which may have introduced selection and recall bias. Many of the studies use subjective measures to quantify the frequency and intensity of exercise, incorporating reporting bias. The lack of standard definitions of exercise intensity and the wide variability of exercise undertaken by participants into these studies introduce significant complexity to the meta-analysis and the clouds interpretation of the results. Populations studied differ in terms of ethnicity, age, and gender. In some prospective studies which have examined the effect of exercise on various cardiovascular endpoints [38], an inverse relationship between physical activity and risk of coronary heart disease has been demonstrated; however, the relationship between exercise and stroke risk has been less substantiated. It is likely that this is, in part, due to the lower incidence of cerebrovascular events over the study period. Finally, the majority of the studies did not apply repeated measures of physical activity, and many were underpowered to detect significant associations.

A meta-analysis by Lee et al. included 18 cohort and 5 case control studies and concluded that moderately and high physically active individuals have lower stroke incidence and mortality. The relative reduction in stroke incidence observed in moderately and highly active individuals was estimated at between 20 and 27% [39]. 

Further evidence in support of a beneficial effect of exercise on stroke risk can be found in a meta-analysis of observational data from cohort and case control studies which investigated the effects of occupational and leisure-time physical activity on the risk of stroke [40]. The findings of the meta-analysis were that a high level of occupational physical activity was associated with a stroke risk reduction of 43% when compared to occupational inactivity and a relative stroke risk reduction of 23% when compared to a moderate level of occupational activity. Moderate amounts of physical activity at work were associated with a 36% reduction in risk of stroke compared with being inactive at work. High levels of leisure-time physical activity were associated with a stroke risk reduction of 20–25% when compared to being inactive during leisure time. The risk reduction when comparing moderate levels of leisure-time physical activity to inactivity was 15%. 

Findings from further studies have been broadly supportive of the evidence that physical activity has an independent protective effect on the risk of cerebrovascular events [41–46]. However, it appears that, within the topic of exercise in the primary prevention of stroke, there are issues which remain unresolved: the possibility of a gender-dependent difference in risk reduction, dubiety surrounding the intensities of exercise which are most beneficial, and lack of knowledge of the duration of the cerebrovascular benefits of exercise.

### 4.1. The Effect of Gender

A meta-analysis, including data from 33 prospective cohort studies and 10 case control studies addressing the effects of physical activity on stroke-related morbidity and mortality, found that the risk of an ischaemic stroke was reduced by 24% in women and 27% in men. However, they reported the effect to be statistically significant only for men [41]. This finding was supported by a further prospective study [46] but has not been reproduced in several large studies assessing the effect of exercise in females which have suggested benefit [43, 44]. 

A prospective cohort study of participants in the Women's Health Study [47] investigated the effect of different lifestyle components on stroke risk in women, found a weak association between risk of stroke and physical activity alone but combining physical activity with smoking cessation, low BMI, healthy diet and moderate alcohol consumption was associated with a significant reduction in stroke risk [43].

More recently, Sattelmair et al. investigated the effect of physical activity on stroke risk in 39315 women from the Women's Health Study (WHS) with a mean followup of 11.9 years [44]. They observed inverse associations of borderline significance between leisure-time physical activity and risk of stroke, with a 17% stroke risk reduction being observed for the most active women in comparison to the least active women in the study. In their report, they compared their results to those of a Japanese cohort [45], where a risk reduction of 17% (for fatal stroke only) was also observed, and to studies in Finland [29], Norway [18], and the United States [20] where risk reductions of 34%, 53% (for fatal stroke only), and 25%, respectively, were reported.

Many of the studies have not included women; therefore, there is less evidence on exercise in the prevention of stroke for women from which to draw conclusions and formulate guidelines and recommendations. 

### 4.2. Intensity of Exercise

The Northern Manhattan Stroke Study [25], a retrospective case control study that found leisure-time physical activity to be protective against ischaemic stroke, was followed recently by a prospective study in the same community [46]. The findings were that engaging in moderate to high intensities of physical activity, such as jogging, swimming, or tennis, was associated with a lower risk of ischaemic stroke; however, light activity (such as walking) did not confer the same benefit. The protective effect was seen only in men. This is in contrast to the findings of the Women's Health Initiative [47] and the Nurses' Health Study [20], where mild intensity physical activity, such as walking, was associated with a reduced risk of stroke. 

### 4.3. Duration of Effect

In order to maintain the cardiovascular benefits and stroke risk reduction associated with exercising, patients must continue to participate in regular physical activity. If indeed a large proportion of the benefit of exercise on stroke risk reduction is mediated through its effect on reducing blood pressure, as some have suggested, then it is important to note that the positive effect of blood pressure reduction is reversible with cessation of exercise [48]. A recent case control study found no evidence of a protective effect of sporting activity during young adulthood against stroke and TIA in later life [49].

Evidence suggests that regular physical activity reduces the incidence and the mortality associated with stroke. The relative risk reduction appears to be somewhere between 20 and 30%. Some studies, though not all, report a trend for higher risk reduction with higher intensity physical activity [25, 30, 32, 39, 46]. However, a recurring problem is that definitions of low, moderate, and high intensity physical activity vary widely between studies, and many of the trials have used self-reported physical activity questionnaires to assess levels of physical activity, which tend to be imprecise and biased with large measurement error. It is also clear that the degree of control over confounding variables has also varied among studies accounting for differences in values of risk reduction. Some trials have failed to demonstrate a significant reduction in stroke risk in women despite evidence of benefits in men. Possible reasons for this include smaller numbers of women being included in trials; therefore, studies are often underpowered to detect a significant effect, or confounders, which remain unaccounted for such as differences in diet or hormonal therapies, could be responsible for masking associations between physical activity and stroke risk in women.

In summary, published evidence favours an association between exercise and cardiovascular health. Beneficial effects of exercise on cerebrovascular risk reduction seem likely; however, definitive controlled trials are justified to establish the intensity and the frequency of exercise required to achieve benefits.

## 5. Physical Activity in Prevention of Recurrent Stroke

Approximately 30% of strokes are recurrent in nature [50]. Stroke and acute cardiac events have a higher incidence in patients with previous stroke than in the general population [51]. The American Heart Association's 2004 recommendations for Physical Activity in Stroke Survivors encourages targeting modifiable risk factors, such as physical inactivity, to decrease the frequency of recurrent events. At present there is very limited long-term followup available examining physical activity levels and recurrent events in stroke survivors. Extrapolation of the effects of physical activity on cardiovascular risk factor reduction and physical fitness in the nonstroke population predicts that regular exercise delivered through stroke rehabilitation programmes may reduce the risk of further cerebrovascular and cardiovascular events in stroke survivors, subsequently reducing the risk of mortality in this population. A preliminary study, examining the effects of three different exercise interventions on cardiorespiratory fitness and coronary risk reduction in stroke survivors, found that 30 minutes of moderate-intensity aerobic exercise was more effective than 60 minutes of lower-intensity aerobic exercise or nonaerobic therapeutic exercise in reducing blood pressure and blood lipid levels [52]. There is a general consensus that a potential stroke risk reduction may exist for stroke survivors who participate in regular physical activity; however, this is based largely upon the effect of exercise upon surrogate markers of stroke risk, and prospective clinical endpoint studies are lacking.

## 6. Effect of Physical Activity on Functional Outcome after Stroke

With recent improvements in the care of patients presenting with an acute stroke and wider availability of pharmacological treatments, the majority of patients are surviving the initial insult [53]. Despite this, only 1 in 3 patients with stroke makes a full recovery, and significant healthcare resource is consumed in the care of patients with stroke [54]. The evidence base for rehabilitation strategies after stroke is relatively weaker than acute treatments [55], and further work is needed to optimise rehabilitation, minimise impairment, and improve function in stroke survivors.

Physical fitness is greatly reduced in people after stroke when compared to their age-matched counterparts [56], and stroke sufferers with residual disability are less likely to exercise regularly [57, 58]. This is predictable as many stroke survivors are left with residual impairments such as reduced mobility, poor balance, and decreased muscle strength making physical activity more challenging and a sedentary lifestyle more likely. Clinical guidelines for the management of stroke recommend physical activity as part of the rehabilitation process [1, 3] as evidence suggests that it can improve physical fitness and reduce the impact of stroke-induced disability [59]. Reducing disability can improve independence and overall quality of life [60].

The American Heart Association's scientific statement from 2004 concluded that training-induced cardiovascular health and fitness benefits seen in the general population may be extrapolated to stroke survivors [3]. This was based on evidence from five separate studies which collectively demonstrated improvements in peak oxygen consumption, submaximal energy expenditure, resting heart rate, peak left ventricular ejection fraction, aerobic capacity, and overall exercise capacity in stroke survivors undertaking aerobic exercise training [61–65]. This is consistent with data from studies involving both stroke and nonstroke patients, where benefits of regular physical activity in reducing multiple cardiovascular risk factors have been highlighted [6, 25, 66–68].

A meta-analysis assessing the effect of aerobic training on aerobic capacity by reviewing results from nine articles (seven randomised controlled trials) found that aerobic training achieving 50–80% of heart rate reserve, for 20–40 minutes, 3–5 days per week results in improved aerobic capacity as determined by peak oxygen consumption and peak workload. Most of the studies involved in the meta-analysis used the treadmill, cycle ergometer, or functional activities such as brisk stepping as a mode of aerobic training, and the analysis did not find that combining aerobic with resistance training resulted in improvements in aerobic capacity in this poststroke population [69].

Current stroke rehabilitation guidelines [1, 3] are generally centred around physiotherapy and occupational therapy regimens and are often focused on decreasing disability from stroke-induced impairment, to encourage patient independence as much as possible. The extent these programmes improve aerobic fitness is unclear, and experimental data have been inconsistent. One study found that rehabilitation programmes induced target heart rates in stroke patients suggesting the possibility of a training effect from functional exercise programmes [70]. However, another study evaluating the cardiovascular stress generated by physiotherapy and occupational therapy interventions in twenty patients enrolled in a stroke rehabilitation programme found that patients participating did not spend a large enough proportion of each session in their target heart rate zone and, therefore, were failing to generate an adequate cardiovascular stress to produce a training effect [71]. In addition, it is recognised that stroke rehabilitation programmes vary considerably between different centres, and; therefore, higher heart rates and prolonged durations of exertional tachycardia may occur with some programmes and not with others. 

The loss or reduction in motor function is the most common and widely recognized impairment resulting from stroke [53]. Many studies investigating the benefits of exercise in stroke survivors have evaluated the benefit of physical therapy in improving function: strength, gait, and balance. 

Previous advice to avoid strength training in stroke patients in order to decrease the chance of developing spasticity has not been substantiated [55]. On the contrary, the evidence for resistance training to improve muscle strength is abundant [64, 72–76] with a meta-analysis reporting a dose response relationship [77]. 

A recent meta-analysis by Langhorne et al. reported on a wide range of interventions that have been shown to improve motor function after stroke [53]. Upper limb functioning has been shown to improve significantly with the use of the following techniques: constraint-induced movement therapy (CIMT) [78], a form of physiotherapy where repetitive tasks are performed with the paretic limb, EMG biofeedback [79, 80] where electrodes are applied to the muscles to report electrical potentials to the patient via an auditory or visual means, mental imagery [81, 82] where physical functions are repeatedly mentally rehearsed, and robotics [83–85] which allow high-intensity repetitive movements of the upper limb to be carried out. A borderline effect was also observed in upper limb functioning with repetitive task training and electrostimulation [2] and bilateral movement training to improve control, and movement of the paretic limb has also shown favourable results in a meta-analysis [86]. 

The Copenhagen Stroke Study reported that 22% of stroke survivors are unable to walk at the end of rehabilitation programmes [87]. Independent gait is closely related to independence and achievement of activities of daily living and, therefore, is one of the aims of physical training after stroke. A systematic review investigating the effectiveness of lower-limb strengthening, cardiorespiratory or gait-oriented tasks in improving gait, gait-related activities, and health-related quality of life in stroke survivors evaluated 21 randomised controlled trials. The evidence generated supports the conclusion that gait-oriented training improves walking competency after stroke but has no significant effect on activities of daily living or health-related quality of life [88]. A previously mentioned review examined the effects of cardiorespiratory with strength training, high-intensity physiotherapy, and repetitive task training on gait and found that only cardiorespiratory training is supported by strong evidence of beneficial effects on walking ability [53].

There is limited evidence that the use of technological devices improve gait in stroke patients. The use of electromechanical gait training to improve walking after stroke was evaluated in a systematic review of seventeen randomised controlled trials. Results indicated that electromechanical-assisted gait training in combination with physiotherapy after stroke enhances patients' chances of achieving independent walking. No benefits were found to walking speed or endurance [89]. Treadmill training was not found to be efficacious in improving walking in patients after stroke [90]; however, in a subgroup analysis, a trend towards better outcomes was demonstrated in patients using treadmill training with body weight support.

Biofeedback [91–94], where the patient is given information about his/her position and weight distribution using a force platform, and repetitive task training [95, 96] are the most frequently used interventions for improving balance in stroke survivors. However, neither intervention is supported by robust evidence of its effectiveness [53].

Participation in regular physical activity can be beneficial to patients who have had a stroke. Aerobic training has been shown to enhance physical fitness and reduce cardiovascular risk factors in stroke patients who are generally less physically active than age-matched counterparts, and physical activity guidelines now recommend dedicating more time to aerobic activity as part of stroke rehabilitation programmes to optimise cardiovascular and cerebrovascular benefits and reduce the risk of recurrent events [3]. Various trials have investigated the use of exercise and physical activity to improve strength, gait, and balance in patients with stroke. There is strong evidence to support the use of resistance training to improve muscle strength, and gait-oriented and cardiorespiratory training have shown potential benefits in improving gait and walking ability. Difficulties arise in providing guidelines for exercise recommendation in patients with stroke, as an individual's ability to exercise will vary with stroke subtype, residual disability, age, and co-morbidity. Further detail regarding types of exercise and the optimal intensities and frequencies at which they should be performed should be sought in order to utilise the potential benefits of exercise in stroke patients. 

Despite the potential benefits, in reality the implementation of exercise for stroke patients is complex; there are often significant barriers to exercise prescription. Clearly the nature and severity of the neurological deficit will influence the range of activities deemed suitable for the patient. Further, previous studies have highlighted that, even in the optimal setting of an acute stroke unit, the time devoted to each individual's physical and occupational therapy is limited by economic constraints, such as limited resources and personnel. As stroke is predominantly a disease of the elderly, many patients have significant comorbidities which may make regular physical activity less feasible [97, 98]. A meta-analysis by Kwakkel et al. suggests that greater intensity and frequency of therapy improve outcomes [98]; however, observed differences were modest and perhaps not worth the associated expense. Low mood and reduced self-esteem in the poststroke period could perhaps also contribute to the decreased motivation of patient to participate in exercise, especially group exercise. Finally, although a dose response relationship may exist between outcome and exercise, adverse events such as musculoskeletal injuries or sudden cardiac death is a potential threat. Guidelines encourage a preexercise evaluation of stroke patients to assess whether participation in exercise is suitable; the benefits outweigh the risks [3].

Further trials are required to provide robust evidence to enable the design of exercise regimens, which can be implemented into stroke rehabilitation programmes to maximise functional outcomes in patients after stroke.

## 7. Conclusion

Exercise and physical activity are useful tools in the rehabilitation and the functional recovery of patients who have suffered a stroke. In addition, physical activity potentially provides protective benefits in the prevention of stroke, which may extend beyond the positive effects on traditional cardiovascular risk factors.

Based on the available evidence, the American Heart Association (AHA) recommends that stroke survivors should undertake: strength training to increase independence in activities of daily living, flexibility training to increase range of movement and prevent deformities, and training to enhance balance and coordination. The AHA advises that each of these exercise modalities should be carried out twice or three times per week with the view to improving functional outcome after stroke. Aerobic exercise of moderate intensity should be carried out on at least three days of the week for twenty to sixty minutes at a time, in order to increase physical activity capacity, improve walking and independence, and reduce the risk of cardiovascular disease [3]. This guidance is derived, at least in part from the extrapolation of data from other non-stroke populations. 

Limitations highlighted in previous studies which have investigated the benefits of exercise in the prevention of stroke inhibit definitive conclusions regarding the type, frequency, and intensity of exercise that is required to confer a protective effect. Although some trials have included data on haemorrhagic stroke, the main body of evidence is for ischaemic stroke, and data on physical activity and haemorrhagic stroke are lacking in comparison. Profitable topics for further investigation should focus on defining the optimal intensities and durations of exercise required to provide the most substantial reduction in stroke risk for use in both primary and secondary prevention, the frequency of exercise sessions, the effect of gender on risk reduction with exercise, and the duration of observed benefit. For patients with previous stroke, identification and targeting of barriers to exercise delivery could lead to more widespread implementation of exercise prescription in this population. The long-term effect of regular physical activity on recurrent stroke risk in patients with previous stroke merits further study.

## Figures and Tables

**Table 1:** Characteristics of studies investigating physical activity and stroke risk.

Study	Year	Methodology	Number of participants	Population Characteristics	Followup (years)	Number of stroke events	Relationship between physical activity and stroke
Abott et al. [16]	1994	Prospective cohort	7530	Male (Honolulu Heart Program) 55–68 years	22	537	Inverse association
Agnarsson et al. [17]	1999	Prospective cohort	4484	Male (The Reykjavik Study) 45–80 years	10.6	249	Inverse association
Ellekjær et al. [18]	2000	Prospective cohort	14101	Female (Nord-Trondelag Health Survery) ≥50 years	10	457 stroke deaths	Inverse association
Evenson et al. [19]	1999	Prospective cohort	14575	Male and Female (The Atherosclerosis Risk in Communities Study) 45–64 years	7.2	189	Inverse association
Hu et al. [20]	2000	Prospective cohort	72488	Female (The Nurses' Health Study) 40–65 years	8	407	Inverse association
Kiely et al. [21]	1994	Prospective cohort	4196	Male and female (The Framingham Study) 28–62 years	32	427	Inverse association
Gillum et al. [22]	1996	Prospective cohort	7895	Male and female (National Health and Nutrition Examination Survey I) 45–74 years	11.6	623	Inverse association
Haheim et al. [23]	1993	Prospective cohort	14403	Male (Oslo Study) 40–49 years	12	81	Inverse association
Wannamethee and Shaper [24]	1992	Prospective cohort	7735	Male (British Regional Heart Study) 40–59 years	9.5	128	Inverse association
Sacco et al. [25]	1998	Retrospective case control	369 + 678 controls	Male and Female ≥39 years 1st ever cerebral infarction	—	—	Inverse association
Shinton and Sagar [26]	1993	Retrospective case control	65 + 169 controls	Male and female 35–74 years 1st ever stroke and no significant comorbidities	—	—	Inverse association
You et al. [27]	1995	Retrospective case control	203 + 203 controls	Male and female 20–85 years Lacunar infarct	—	—	Inverse association
You et al. [28]	1997	Retrospective case control	201 + 201 controls	Male and female 15–55 years Cerebral infarct	—	—	Inverse association
Hu et al. [29]	2005	Prospective cohort	47721	Male and female 25–64 years	19	2863	Inverse association
Williams et al. [30]	2009	Prospective cohort	41402	Male and female (National Runners' Health Study)	7.7	119	Inverse association
Lee et al. [32]	1999	Prospective cohort	21823	Male (Physicians Health Study) 40–84 years	11.1	533 stroke deaths	No clear association
Lee and Paffenbarger Jr. [33]	1998	Prospective cohort	11130	Male (The Harvard Alumni Health Study) 43–88 years	13	378 stroke deaths	U-shaped association
Fossum et al. [34]	2007	Prospective cohort	9193	Male and female Left ventricular hypertrophy and hypertension patients (Losartan intervention for endpoint reduction in hypertension (LIFE) study) 55–80 years	4.8	541 strokes	Inverse association
Lindsted et al. [35]	1991	Prospective cohort	9484	Male (The Seventh Day Adventist Study) ≥30 years	26	410 stroke deaths	No inverse association
Menotti and Seccareccia [36]	1985	Prospective cohort	99029	Male (Italian Railroad Workers Study) 40–59 years	5	187	No clear association
Simonsick et al. [37]	1993	Prospective cohort	4840	Male and female (Established Populations for Epidemiologic Studies of the Elderly) ≥65 years	6	—	Inconsistent relationships between physical activity and stroke

Key: — = Data not available.
